# Protective Effects of *Caesalpinia sappan* Linn. and Its Bioactive Compounds on Cardiovascular Organs

**DOI:** 10.3389/fphar.2021.725745

**Published:** 2021-09-15

**Authors:** Mas Rizky AA Syamsunarno, Ratu Safitri, Yusof Kamisah

**Affiliations:** ^1^ Department of Biomedical Sciences, Faculty of Medicine, Universitas Padjadjaran, Jatinangor, Indonesia; ^2^ Faculty of Mathematics and Natural Sciences, Universitas Padjadjaran, Jatinangor, Indonesia; ^3^ Department of Pharmacology, Faculty of Medicine, Universiti Kebangsaan Malaysia, Kuala Lumpur, Malaysia

**Keywords:** *Caesalpinia sappan*, brazilin, sappanone A, brazilein, ischemia/reperfusion injury, vasorelaxation, heart, vascular

## Abstract

Cardiovascular diseases are the leading cause of death worldwide. The long-term aim of cardiovascular disease therapy is to reduce the mortality rate and decelerate the progression of cardiovascular organ damage. Current therapies focus on recovering heart function and reducing risk factors such as hyperglycemia and dyslipidemia. However, oxidative stress and inflammation are important causes of further damage to cardiovascular organs. *Caesalpinia sappan* Linn. (Fabaceae), a flowering tree native to tropical Asia, has antioxidant and anti-inflammatory properties. It is used as a natural dye to color food and beverages and as a traditional treatment for diarrhea, diabetes, and blood stasis. The phytochemical compounds in *C. sappan*, mainly the homoisoflavonoids brazilin, sappanone A, protosappanin, and hematoxylin, can potentially be used to protect cardiovascular organs. This review aims to provide updates on recent developments in research on *C. sappan* in relation to treatment of cardiovascular diseases. Many studies have reported protective effects of the plant’s bioactive compounds that reduce cardiac damage and enhance vasorelaxation. For example, brazilin and sappanone A have an impact on molecular and cellular changes in cardiovascular disease pathogenesis, mainly by modulating oxidative, inflammatory, and apoptotic signaling pathways. Therefore, bioactive compounds of *C. sappan* have the potential to be developed as therapeutic agents to combat cardiovascular diseases like myocardial infarction and vascular disease. This review could help further the understanding of the possible modulatory role of the compounds in cardiovascular diseases, thereby facilitating future studies.

## Introduction

Cardiovascular disease is a leading cause of morbidity and mortality globally, with heart attack and stroke accounting for about 85% of these deaths, the majority occurring in middle- and low-income countries (WHO, 2017). Cardiovascular disease includes disorders of the heart and blood vessels, such as ischemic heart attack or myocardial infarction, heart failure, hypertension, and cerebrovascular disease (Leong et al., 2017). The pathogenesis of these disorders involves oxidative stress and inflammation (Siti et al., 2015).

Therefore, intervention with medicinal plants possessing antioxidant and anti-inflammatory properties may alleviate the severity of the disease. Numerous studies have investigated medicinal plants for their potential pharmacological activities in cardiovascular organs, and many have shown promising effects. *Parkia speciosa* Hassk. alleviated cardiac damage in hypertensive rats (Kamisah et al., 2017), *Hibiscus sabdariffa* L. showed cardioprotective effects in myocardial infarction-induced rats (Si et al., 2019), and *Caesalpinia sappan* Linn. extract demonstrated a vasorelaxant effect on rat arteries (Sasaki et al., 2010).

*Caesalpinia sappan* is a medicinal plant that possesses antioxidant (Suwan et al., 2018) and anti-inflammatory (Tewtrakul et al., 2015) properties. It exerts protective effects on the cardiac (Nugraheni and Saputri, 2017) and vascular (Sasaki et al., 2010) systems. Investigations of this plant have progressed to isolation of the active metabolites, such as brazilin, sappanchalcone, and protosappanin D (Sasaki et al., 2010), that might be responsible for the protective effects, but these studies are still at the initial phase. Therefore, this review aims to gather information on recent updates to studies on *C. sappan* extract and the effects of its metabolites on cardiovascular organs. Our findings could accelerate future research on the plant and the development of its metabolites as alternatives to modern medicine.

## *Caesalpinia sappan* and its Bioactive Metabolites

*Caesalpinia sappan* Linn. (synonymous with *Biancaea sappan*), commonly known as sappanwood, grows abundantly in Southeast Asia, southern China (Li et al., 2020) and the Indian subcontinent, either in the wild or as a cultivated tree (Mariappan et al., 2014). It is known locally as secang or sekang in Indonesia, *pokok sepang* in Malaysia (Nugraheni and Saputri, 2017), and pattanga in India (Chellappan et al., 2017). The plant belongs to the family Fabaceae (subfamily Caesalpinioideae), and is a shrubby tree that grows to 10 m tall, with a ca. 14 cm diameter trunk and alternate bipinnate leaves (Mariappan et al., 2014). It bears clusters of flat, oblong pods that contain brown flattened and ellipsoid seeds (Warriers et al., 1993). Its wood is hard and orange red. Its heartwood has been traditionally used to treat bleeding gums, anemia, diabetes, cardiac problem and blood stasis and as a post-partum tonic to reduce uterine bleeding; it is also known for its antidiarrheal, sedative, and diuretic properties (Badami et al., 2004; Mekala and Radha, 2015; Li et al., 2020). In Vietnam, *C. sappan* is used to decrease the symptoms of rheumatism and inflammatory diseases (Do, 2001). It is also an ingredient in several Ayurvedic preparations (Mekala and Radha, 2015).

**FIGURE 1:**
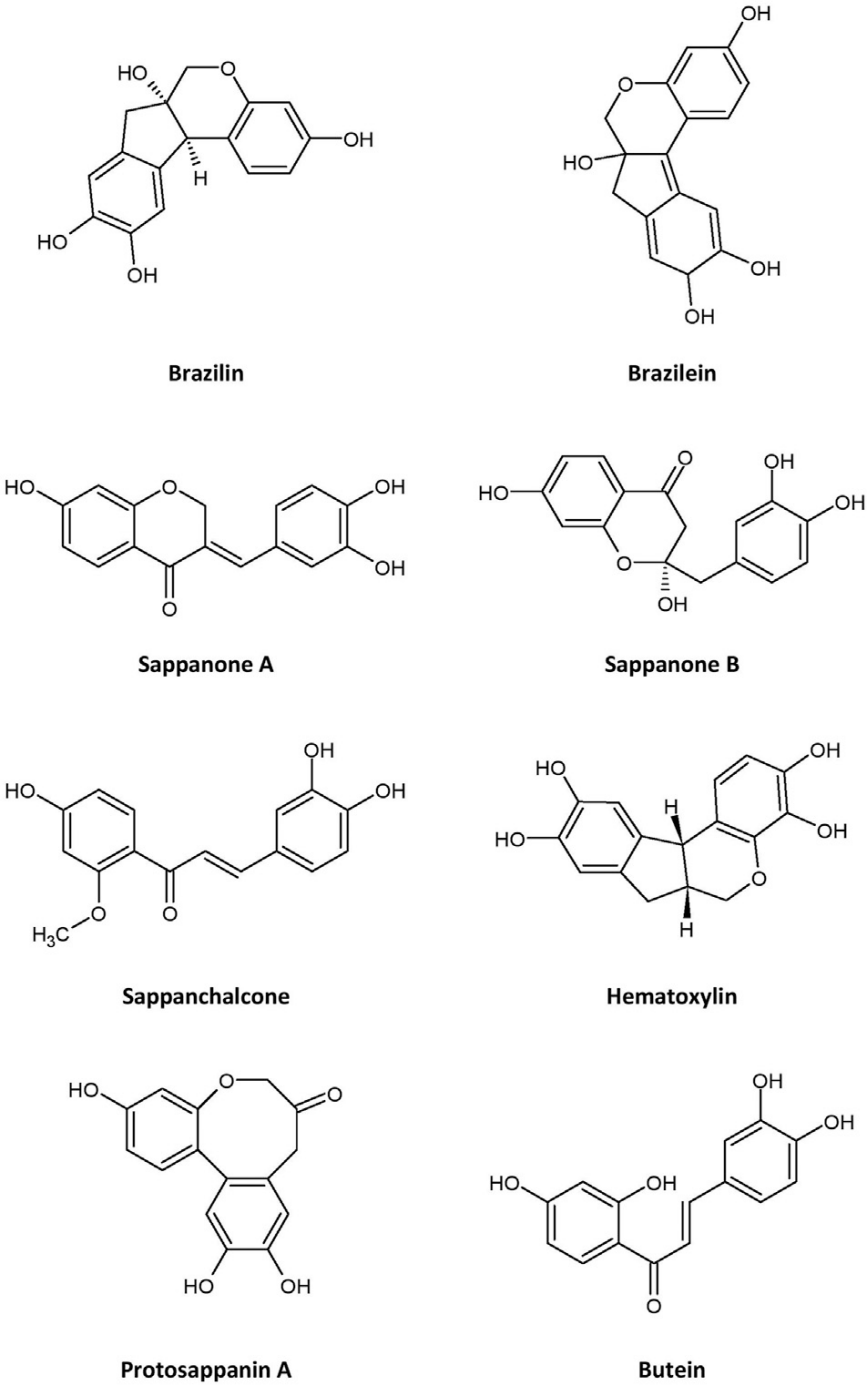
Chemical structures of major metabolites isolated from *Caesalpinia sappan*.

Many bioactive compounds have been isolated from *C. sappan*. Some of the most abundant phytochemicals present in the plant are homoisoflavonoids, of which brazilin—a natural red dye—is the major active compound (Settharaksa et al., 2019; Uddin et al., 2015), along with its oxidized form, brazilein (Dapson and Bain, 2015), in the heartwood. Other homoisoflavonoids present in the plant are sappanol, episappanol, protosappanin B and C (Mueller et al., 2016; Uddin et al., 2015), caesappin A and B (Wang et al., 2014), sappanone A (Zhao et al., 2020), sappanone B, (E)-3-(3,4-dihydroxybenzylidene)-7-hydroxychroman-4-one (He et al., 2009), deoxysappanone B (Zeng et al., 2015), neosappanone A, neoprotosappanin (Nguyen et al., 2005), and caesalpin P and J (Shimokawa et al., 1985) (Table 1). The chemical structures of the key metabolites in *C. sappan* are presented in Figure 1. These homoisoflavonoids have antioxidant (Uddin et al., 2015), antibacterial (Settharaksa et al., 2019), anti-inflammatory (Choo et al., 2017) and neuroprotective (Zeng et al., 2015) properties. The heartwood also contains the following phenols: caesalpiniaphenol A−H (Cuong et al., 2012; Min et al., 2012; Hung et al., 2013), epicaesalpin J, and 7,10,11-trihydroxydracaenone (Zhao et al., 2014). The latter two compounds do not show significant inhibitory activity against nitric oxide (Zhao et al., 2014), a vasodilator found in blood vessels. The seeds of *C. sappan* are rich in diterpenoids, including caesalsappanin A−N, R and S (Ma et al., 2015; Bao et al., 2016; Zhu et al., 2017; Wang et al., 2020); phanginin A−K and R‒T (Yodsaoue et al., 2008; Bao et al., 2016); and ester glycosides, namely caesateroside A−C (Wang et al., 2020). Oleanolic acid, a triterpenoid, has also been isolated from the plant (Zheng et al., 2020). These diterpenoids exhibit antiplasmodial (Zhu et al., 2017) and antitumor (Bao et al., 2016; Wang et al., 2020) activity.

**TABLE 1:** Phytochemicals isolated from *Caesalpinia sappan*.

Parts of plant	Type	Compound	References
Heartwood	Homoisoflavonoids	Brazilin	Settharaksa et al. (2019)
		Brazilein	Dapson and Bain, (2015)
		Sappanol	Uddin et al. (2015)
		Episappanol	Mueller et al. (2016)
		Protosappanin B−C	Mueller et al. (2016)
		Caesappin A−B	Wang et al. (2014)
		Sappanone A	Zhao et al. (2020)
		Deoxysappanone B	Zeng et al. (2015)
		Neosappanone A	Nguyen et al. (2005)
		Neoprotosappanin	Nguyen et al. (2005)
		Sappanone B	He et al. (2009)
		(E)-3-(3,4-dihydroxybenzylidene)-7-hydroxychroman-4-one	He et al. (2009)
		3′-Deoxy-4-O-methylepisappanol	Fu et al. (2008)
		Caesalpin J and P	Shimokawa et al. (1985)
		Hematoxylin	Xie et al. (2000)
	Phenols	Caesalpiniaphenol A−F	Cuong et al. (2012)
		Caesalpiniaphenol G−H	Hung et al. (2013)
		Epicaesalpin J	Zhao et al. (2014)
		7,10,11-Trihydroxydraca-enone	Zhao et al. (2014)
Seeds	Diterpenoids	Caesalsappanin A−L	Ma et al. (2015)
		Caesalsappanin M−N	Bao et al. (2016)
		Caesalsappanin R and S	Zhu et al. (2017)
		Phanginins A−K	Yodsaoue et al. (2008)
		Phanginins R−T	Bao et al. (2016)
	Triterpenoid	Oleanolic acid	Zheng et al. (2020)
	Ester glycosides	Caesateroside A−C	Wang et al. (2020b)

## Pharmacokinetics and Toxicity of *Caesalpinia sappan* and Its Bioactive Compounds

### Pharmacokinetics

Studies on the pharmacokinetic properties of the *C. sappan* metabolites are still lacking. Only brazilin has been studied extensively. Oral and intravenous administration of brazilin resulted in the incorporation of a similar amount of the phytochemical into the plasma (Jia et al., 2013), indicating its almost complete oral absorption. When brazilin was injected into the tail vein of rats at 50 mg/kg body weight, the plasma area under the curve (AUC) showed that it was absorbed at approximately 1,500 ng h/mL. It has a half-life (t_½_) of 4.4 h, a peak plasma concentration (C_max_) of approximately 1,600 ng/ml, and a time to reach maximum concentration (T_max_) of approximately 2 min (Jia et al., 2013), which suggests a rapid absorption process. Brazilin also demonstrated linear pharmacokinetics in rats, according to the C_max_ and AUC values, which increased with increasing dosage (Yan-yan et al., 2014). On the other hand, when *C. sappan* extract that contained 52.25 mg/kg of brazilin was orally administered at 2.83 g/kg body weight, a similar AUC was observed, but the T_max_ was tenfold longer and the t_½_ was shorter (2.21 h) (Tong et al., 2013) than that of brazilin in a previous study (Jia et al., 2013). This may have been due to other bioactive compounds present in the extract affecting the absorption and/or elimination of brazilin. The compound was dispersed into almost all organs, with the highest concentrations found in the kidneys, followed by the liver and lungs (Jia et al., 2013).

Oral administration of *C. sappan* extract (at 2.83 g/kg body weight) that contained 35.56 mg/kg protosappanin B resulted in a t_½_ and a T_max_ similar to those of brazilin (Tong et al., 2013). Diabetes affected the pharmacokinetics of protosappanin B and brazilin similarly, observed as augmentation of C_max_, AUC, and t_½_. However, diabetes reduced the T_max_ of protosappanin B, but did not affect of that of brazilin (Tong et al., 2013). Taken together, these results suggest that brazilin has an almost complete and fast oral absorption as well as distribution. This property makes the compound a promising candidate for further study.

### Toxicity

A single oral dose of *C. sappan* at 5,000 mg/kg body weight, and repeated at doses of 250, 500 and 1,000 mg/kg for 30 days yielded no toxic effects in male and female Wistar rats. No apparent changes in body weight, the gross appearance of internal organs—the heart, liver, brain, lungs, pancreas, spleen, adrenal glands, kidneys, and sex organs—or general behavior were noted compared to control (Sireeratawong et al., 2010). An aqueous extract of natural dye from *C. sappan* (100–2000 mg/kg) was demonstrated to be safe and did not cause any abnormalities or mortality during 14 days of observation. Similarly, the dye did not have significant subacute toxicity up to 5,000 mg/kg body weight (Athinarayanana et al., 2017). In an *in vitro* study, a *C. sappan* ethanol extract at 10 μg/ml did not significantly reduce the percentage of viable cells with intact morphology in H9c2 cardiomyocytes, but this percentage decreased after the cells were exposed to the extract at 50 μg/ml (Sulistiyorini et al., 2020). These results indicate that the acute administration of *C. sappan* extract in rats is likely safe. However, other toxicity studies, such as chronic and carcinogenic toxicity studies, should be performed to ascertain safety for long-term use. Nevertheless, in the study (Sulistiyorini et al., 2020), the plant was not authentically validated by a botanist, therefore the reproducibility of the findings could not be ascertained.

Previous toxicity studies on the bioactive compounds of *C. sappan* heartwood have only addressed brazilein (Yuan et al., 2016). In a study with ICR mice, brazilein was administered intravenously (at 5, 10, and 20 mg/kg) to non-pregnant females for 14 days and to males for 30 days before mating, and brazilein administration was continued in females after successful mating until 13 days of gestation. This resulted in an increased ratio of resorbed fetuses compared to control, although no deformed fetuses were observed. In addition, the live fetus ratio decreased, and the dead fetus ratio increased after brazilein treatment. No other gravid parameters were affected, and the mating process of the mice was not influenced. At all doses tested, brazilein did not have toxic effects on males, as demonstrated by the weight of the reproductive organs, vitality, and abnormal sperm levels (Yuan et al., 2016). These findings suggest that brazilein should be taken with caution by pregnant women, as it may have a significant impact on embryo development and growth after implantation.

## Effects on Myocardial Injury

### *Caesalpinia sappan* Extract

Only one study to date (Nugraheni and Saputri, 2017) investigated the effects of *C. sappan* crude extract using a myocardial injury model. However, the plant taxonomy could not be confirmed as it was not validated by a botanist. The study investigated the preventive effect of oral *C. sappan* extract as claimed, at doses of 50, 100, and 200 mg/kg for 30 days on isoproterenol-induced myocardial infarction in rats (Table 2). The extract did not reverse the increase in heart weight induced by isoproterenol. However, qualitative observations of the heart infarct area suggested that the extract reduced size or the area with increasing doses. Histologically, the extract significantly reduced myocardial interstitial edema at all experimental doses, and the severity of myocardial necrosis and inflammatory cell infiltration was significantly alleviated at 100 and 200 mg/kg doses of the extract (Nugraheni and Saputri, 2017). These findings demonstrate potential positive effects of *C. sappan* extract that protect against myocardial injury, likely afforded by the presence of bioactive compounds in the extract that was unfortunately not determined in the study. However, in terms of experimental design, the study was not properly outlined with a positive control, which could determine any experimental flaw. It also did not indicate the type of the extract used. An elevation in oxidative stress and inflammation has been reported in isoproterenol-induced myocardial injury (Kumari et al., 2020; Younis et al., 2020). Therefore, the extract most likely elicits protective effects through its antioxidant and anti-inflammatory properties, as previously reported (Tewtrakul et al., 2015; Suwan et al., 2018). The property of the plant extract may support its traditional use in reducing heart problems (Mekala and Radha, 2015). Further *in vivo* studies are necessary to obtain more conclusive evidence of the protective effects of the extract. Heart function should be examined to measure the extent of improvement due to the extract. Other aspects that can be studied are pathways related to myocardial fibrosis, such as the transforming growth factor-β/Smads (TGF-β/Smads) pathway and other pathways linked to oxidative stress, inflammation, apoptosis, and mitogen-activated protein kinase (MAPK) activation.

**TABLE 2:** Effects of *C. sappan* crude extract and its bioactive compounds on myocardial parameters.

	Model	Dose and duration of bioactive compounds	Effects on cardiac parameters			
			Function	Structure	Injury index	Infarct size
Nugraheni and Saputri, (2017)	I/R (*in vivo*)	Crude extract (50, 100 and 200 mg/kg) pretreatment for 30 days	-	↔ HW/BW	-	↓ Infarct size
				All doses		
				↓ interstitial edema		
				100 and 200 mg/kg		
				↓ necrosis		
				↓ inflammatory cell infiltration		
Qi et al. (2021)	I/R (*ex vivo*)	Brazilin (12.5–50 mg/kg, ip) pretreatment for 1 h	↑ LVDP, +dp/dt, −dp/dt	-	↓ CK-MB	↓ Infarct size
			↔ HR		↓ LDH	
Zhao et al. (2006)	*Ex vivo*	Brazilein (0.4–10 mM) concurrently for 2 h	↑ contractility, ↔ HR	-	-	-
			↔ coronary perfusion rate			
Shi et al. (2020b)	I/R (*ex vivo*)	Sappanone A (20 mg/kg, ip) pretreatment for 1 h	↔ HR	-	↓ CK-MB	↓ Infarct size
			↓ +dp/dt, −dp/dt, LVDP		↓ LDH	
Jo et al. (2020)	LAD-induced I/R (*in vivo*)	Sappanone A (50 mg/kg, po) for 5 days (day 0 to day 4 post-I/R)	Day 1	↔ Cardiac structure	↔ CK-MB	↓ Infarct size in distal medial, apex and total region
			↔ EF, FS, E/A, SV, CO, HR, ↑ E′, ↓ E/E′ ↔	↓ Fibrosis in PM	↓ LDH	
			Day 4: ↔ EF, FS, E/A, SV, E′, CO, HR ↓ E/E′	↓ Inflammatory cells in PM and apex	↓ AST	
Qi et al. (2021)	H/R in cardio-myocytes	Brazilin (5–50 µM) pretreatment for 1 h	-	-	↓ CK-MB	-
					↓ LDH	
					↑ cell viability	
					↓ apoptosis	
Shi et al. (2020a)	H/R in cardio-myocytes	Sappanone A (5–50 µM) pretreatment for 1 h	-		↓ CK-MB	
					↓ LDH	
					↑ cell viability	
					↓ apoptosis	
					↓ cTn1	

Abbreviations: AST, Aspartate transaminase; CO, cardiac output; CK-MB, creatine kinase MB; cTn1, cardiac troponin 1; +dp/dt, rate of the rise in left ventricular pressure; −dp/dt; rate of the fall of left ventricular pressure; E′, early relaxation velocity on tissue Doppler; E/A, the ratio of the early (E) to late (A) ventricular filling velocities; E/E′; ratio of transmitral Doppler early filling velocity to tissue Doppler early diastolic mitral annular velocity; FS, fractional shortening; HR, heart rate; ip, intraperitoneum; H/R, hypoxia/reoxygenation; HW/BW, heart weight to body weight ratio; I/R, ischemia/reperfusion; LAD, ligation of the left anterior descending coronary artery; LDH, lactate dehydrogenase; LVDP, Left ventricular developed pressure; po, per oral; PM, papillary muscle; SV, stroke volume; ↔, no change; ↑, increased; ↓, reduced.

Stem extract of *C. sappan* (100 μg/ml) has been found to inhibit phosphodiesterase (PDE) activity in *in vitro*, with ethanol extracts demonstrating greater activity against PDE-1 than hexane and chloroform extracts (Helmi et al., 2020). Although the expression of this enzyme was reported to be elevated in heart failure (Chen et al., 2020), the potential effects of *C. sappan* extract on heart failure have not been studied. Recent evidence suggests that PDE5 and PDE10A inhibition is cardioprotective in patients with systolic heart failure and left ventricular hypertrophy (Lawless et al., 2019; Chen et al., 2020). Inhibition of the enzyme increases the intracellular level of cyclic adenosine monophosphate (cAMP) (Chen et al., 2020), which then boosts Ca^2+^ influx leading to increased myocardial contractility (Irie et al., 2009). Therefore, the extract should be further studied to explore its potential inhibitory regulation of isoenzymes, which could have beneficial effects by improving heart function. The effects of the extract on myocardial contractile function, intracellular calcium concentration, and cAMP should also be explored.

### Brazilin

**FIGURE 2:**
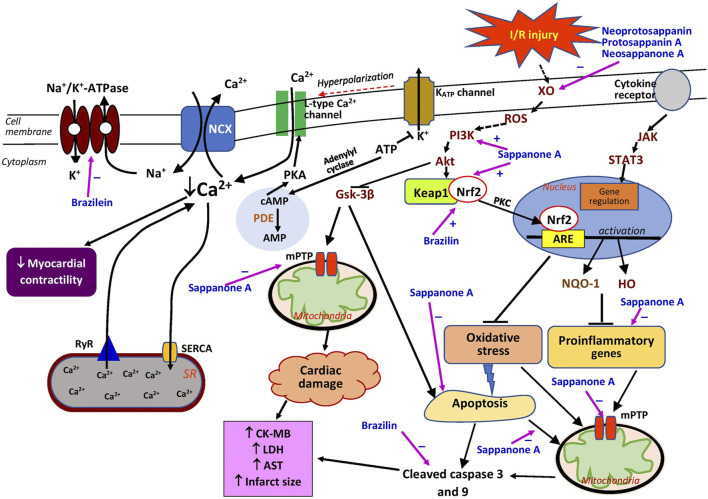
Possible sites of action of *Caesalpinia sappan* bioactive compounds in myocardial injury. Akt, protein kinase B; AMP, adenosine monophosphate; ARE, antioxidant responsive element; AST, aspartate transaminase; ATP, adenosine triphosphate; cAMP, cyclic adenosine monophosphate; CK-MB, creatin kinase MB; GSK-3β, glycogen synthase kinase-3β; HO, heme oxygenase; JAK, Janus kinase; Keap1, Kelch-like ECH-associated protein 1; LDH, lactate dehydrogenase; mPTP, mitochondrial permeability transition pore; Na^+^/K^+^-ATPase, sodium potassium ATPase; NCX, sodium-calcium exchanger; Nrf2, nuclear factor erythroid 2-related factor 2; NQO1, NAD(P)H quinone oxidoreductase 1; PDE, phosphodiesterase; PI3K, phosphatidylinositol 3-kinase; PKA, protein kinase A; PKC, protein kinase C; ROS, reactive oxygen species; RyR, ryanodine receptor; SERCA, sarcoplasmic/endoplasmic reticulum Ca^2+^ ATPase; STAT3, signal transducer and activator of transcription 3; XO, xanthine oxidase; ⊥, suppression; +, stimulates; −, inhibits.

The effects of brazilin on myocardial ischemia/reperfusion (I/R) injury have been investigated through *in vitro* and *ex vivo* models for acute myocardial infarction in humans. In rat cardiomyocytes exposed to hypoxia/reoxygenation (H/R), brazilin (5–50 µM) reduced the release of creatine kinase MB (CK-MB) and lactate dehydrogenase (LDH) in a dose-dependent manner (Table 2). It also decreased H/R-induced apoptosis, observed as a reduction in cleaved caspase 3 (Qi et al., 2021). In the *ex vivo* study, brazilin pretreatment (at 12.5–50 mg/kg intraperitoneally) reduced myocardial infarct size, CK-MB and LDH release, and myocardial apoptosis (at 25 mg/kg) in isolated hearts subjected to I/R injury. It preserved myocardial function by reversing the detrimental effects of I/R on left ventricular (LV) developed pressure (LVDP), the rate of LV pressure increase (+dp/dt), and the rate of LV pressure reduction (−dp/dt) (Qi et al., 2021). No positive control was adopted in both models. Therefore, comparative protective effects with the brazilin-treated group could not be appreciated. The protective effects of brazilin are believed to be mediated by nuclear factor erythroid 2-related factor 2 (Nrf2), a gene involved in the modulation of oxidative stress (Figure 2). Kelch-like ECH-associated protein 1 (Keap1), a substrate adaptor, suppresses Nrf2 transcriptional activity through the formation of a complex between the domains of Nrf2 and Keap1. Upon stimulation of oxidative stress, Nrf2 is dissociated from Keap1 and translocated into the nucleus (Ahmed et al., 2017). Brazilin enhances the nuclear translocation of Nrf2 via the protein kinase C pathway, thus promoting the expression of its target proteins—heme oxygenase-1 (HO-1) and NAD(P)H:quinone oxidoreductase 1(NQO1), which have inhibitory effects on proinflammatory genes (Qi et al., 2021). These findings suggest that brazilin is a promising candidate for protecting against myocardial injury. Additionally, brazilin may confer protection through other mechanisms; for example, it may inhibit the expression of nuclear factor-κB (NF-κB) and its inflammatory signaling pathway. Blockade of NF-κB improved cardiac function and attenuated cardiac remodeling in a myocardial infarct mouse model (Kawano et al., 2006). Brazilin has also been demonstrated to possess PDE-1 inhibitory activity *in vitro* (Helmi et al., 2020), leading to an augmented level of cAMP, which also functions as a fibrotic response modulator (Delaunay et al., 2019). The inhibition of this enzyme led to a decreased fibrotic response in cardiomyocytes (Chen et al., 2020), which may be useful for the therapeutic management of heart failure. However, further studies are needed to confirm this activity *in vivo* and to explore other potentially related mechanisms of brazilin, such as myocardial protein synthesis, fibrosis, autophagy, and hypertrophic signaling.

### Sappanone A

Two studies have investigated the effects of sappanone A on myocardial I/R injury (Shi et al., 2020b; Jo et al., 2020). In rats with ligated left anterior descending coronary artery, oral administration of sappanone A at 50 mg/kg for 5 days starting on the day of ischemia induction, significantly reduced the infarct size, particularly in the distal medial and apical myocardial areas, better than that of curcumin, a positive control (Jo et al., 2020). However, the use of curcumin as the positive control was less appropriate since it is not used clinically for heart diseases. In addition, histological analysis revealed that the homoisoflavonoid treatment diminished inflammatory cell infiltration, with larger effects on lymphocytes in all medial myocardial and epicardial regions. It also reduced fibrosis in papillary muscle, comparable to curcumin (Jo et al., 2020). Furthermore, the results indicated that sappanone A reduced fibrosis in the papillary muscle. The positive effects of the compound were supported by decreases in serum CK-MB, LDH, and aspartate transaminase (Jo et al., 2020), indicators of myocardial injury. Shi et al. (2020b) demonstrated that intraperitoneal administration of sappanone A at 20 mg/kg per hour prior to I/R injury induction exerted protective effects in isolated Langendorff hearts, as indicated by a reduction in the myocardial infarct size and a release of myocardial enzymes (CK-MB and LDH). The cardioprotective effects of the compound could also be attributable to its anti-inflammatory properties (Wang et al., 2021).

Both the above studies demonstrated that sappanone A improved cardiac function after I/R (Jo et al., 2020; Shi et al., 2020b) (Table 2). LVDP, +dp/dt, and −dp/dt increased in the sappanone A-treated group, as measured in isolated hearts (Shi et al., 2020b), suggesting improvement in LV function. In the *in vivo* study (Jo et al., 2020), sappanone A did not significantly affect LV fractional shortening (FS) or the ejection fraction (EF), two indicators of LV systolic function, although a tendency for a reversal effect was observed. Tissue Doppler imaging revealed that the treatment improved the early relaxation velocity (E′), an indicator of LV diastolic function, on day 1 post-I/R induction but not on day 4. Moreover, positive effects of the compound on the ratio of transmitral Doppler early filling velocity to tissue Doppler early diastolic mitral annular velocity (E/E′), another indicator of LV diastolic function, were noted on both day 1 and day 4 (Jo et al., 2020). The findings of these studies strongly suggest that sappanone A from *C. sappan* affords better cardioprotection in the early stage of myocardial infarction, resulting in the mitigation of LV dysfunction. It is possible that the preservation of the cardiac function by sappanone A due to its inhibitory effect on fibrosis in the heart (Jo et al., 2020). However, the detailed mechanisms of the antifibrotic property of the compound have yet to be studied.

Treatment with sappanone A resulted in the alteration of the mRNA expression of 2020 genes, including 66 proinflammatory-related genes believed to be involved in myocardial infarction. Sappanone A may exert positive effects by restoring the genes involved in inflammatory responses. It downregulates the expression of the proinflammatory genes Tgfb1, Tgfb2, Tnfrsf1a, Il18, Pik3cd, Cd4, and Cd8a, as well as the apoptotic gene Casp3, which are activated by myocardial infarction (Jo et al., 2020). In cardiomyocytes, sappanone A alleviated H/R-induced injury by inhibiting mitochondrial apoptosis, observed as repressed caspase-3 and caspase-9 cleavage, leading to increased cell viability. The compound also mitigated mitochondrial permeability transition pore (mPTP) opening and transmembrane potential (Δψm) release (Shi et al., 2020a), possibly because of its antioxidant and anti-inflammatory activities (Figure 2) that reduce reactive oxygen species (ROS), which stimulate the opening of mPTPs, increase the permeability of mitochondria, and subsequently rupture the organelles (Zhang et al., 2016). Sappanone A is reported to provide cardioprotection against I/R-induced injury by activating the phosphatidylinositol 3-kinase/protein kinase B/glycogen synthase kinase-3β (PI3K/Akt/GSK-3β) signaling pathway without affecting the survivor activating factor enhancement (SAFE) pathway, which can be explained by its lack of effect on signal transducer and activator of transcription 3 (STAT3) phosphorylation in cardiomyocytes (Shi et al., 2020a). As a substitute pathway for cardioprotection against I/R injury, the latter pathway is involved in promoting cardiomyocyte survival (Hadebe et al., 2018). The effects of sappanone A on the activation of Akt and Gsk-3β have also been reported in PC-12 cells obtained from pheochromocytomas of the adrenal glands (Kang et al., 2019). The PI3K/Akt/GSK-3β signaling pathway is activated by an increased ROS level which is partly due to the increased activity of xanthine oxidase (Figure 2). The activation of the pathway leads to inhibition of mitochondrial apoptosis and mPTP opening, resulting in decreased cardiac damage. Sappanone A also activates the Keap1/Nrf2 signaling pathway (Shi et al., 2020b), which is one of the crucial signaling pathways controlling the activity of Nrf2, a protein that regulates antioxidant proteins (Tu et al., 2019). Together, these effects suggest that sappanone A could be a potential therapeutic agent for alleviating myocardial I/R injury by targeting mitochondria through the mitigation of the inflammatory, oxidative stress, and apoptosis signaling pathways.

### Brazilein

At 0.4–10 mM concentrations, brazilein had a concentration-dependent cardiotonic effect with negligible impacts on coronary perfusion and heart rate in normal isolated guinea pig hearts, better than its positive control, noradrenaline (30 μM); this effect may not involve the stimulation of the β-adrenoceptor because the addition of propranolol, a β-adrenoceptor blocker, had no effect (Zhao et al., 2006). However, the concentrations used were larger than the ones proposed (30–50 μM) for *in vitro* studies (Heinrich et al., 2020). It is quite difficult to translate high concentrations of pure compounds employed *in vitro* for a therapeutic use in humans, as it may pose toxicity.

Brazilein inhibited Na^+^/K^+^-ATPase (the sodium–potassium pump) (Figure 2) but this effect was not modified by increasing concentrations of potassium (Zhao et al., 2006). This finding suggests that the inhibitory effect of brazilein may not be associated with its binding to E2P (Zhao et al., 2006), which in turn prevents E2P from binding to potassium resulting in the inhibition of the sodium–potassium pump. High concentrations of potassium can reverse the inhibitory effects of cardiac glycosides on the sodium–potassium pump and promote the dissociation of E2P and inhibitors (Kanai et al., 2020), suggesting that brazilein has a different mechanism from that of cardiac glycosides. The sodium–potassium pump indirectly regulates the intracellular calcium level in the heart. Its inhibition elevates the myocardial intracellular calcium level by decreasing the calcium efflux through the Na^+^/Ca^2+^ exchanger, which then increases myocardial contractility (Salim et al., 2020). Brazilein may modulate other calcium regulators, such as ryanodine receptor 2 (RyR2), L-type calcium channel, and sarcoplasmic/endoplasmic reticulum calcium ATPase (SERCA). It was reported that the vasocontraction effects of brazilein depend on the extracellular calcium level (Shen et al., 2008). In terms of its toxicity, brazilein (at 4–48 mg/kg) is less likely to cause cardiac arrythmias than deslanoside (at 400–560 μg/kg), a sodium–potassium pump inhibitor (Zhao et al., 2006), implying a higher therapeutic index for the former. Therefore, brazilein has the potential to be developed as an inotropic drug with Na^+^/K^+^-ATPase inhibition as the therapeutic target. Its effects on other calcium regulatory proteins—Na^+^/Ca^2+^ exchanger, RyR2, and SERCA—should also be studied.

### Other Compounds

The bioactive compounds, neoprotosappanin, protosappanin A, protosappanin A dimethyl acetal, protosappanin E-2, neosappanone A, sappanol, deoxysappanone B, sappanone B, and sappanchalcone isolated from *C. sappan* extract and its methanol ethyl acetate fraction have been reported to possess xanthine oxidase-inhibiting activity (Nguyen et al., 2004, 2005), which could be beneficial in alleviating myocardial injury. Among these compounds, sappanchalcone demonstrated the most potent activity, comparable to allopurinol (Nguyen et al., 2004, 2005). The protective effect is most likely due to these compounds’ antioxidant properties. Previous research indicated that some of these compounds possess antioxidant properties (Sasaki et al., 2007). However, no studies investigating these compounds have considered their effects on the heart. The serum xanthine oxidase level is reported to be elevated in patients with myocardial infarction (Ali et al., 2014). This enzyme produces abundant ROS during cardiac ischemia (Figure 2), which causes further damage to the heart (Bagheri et al., 2016). A meta-analysis of randomized clinical trials reported that purine-like xanthine oxidase inhibitors, such as allopurinol, reduced in the incidence of adverse cardiovascular outcomes (Bredemeier et al., 2018). Further investigations of *C. sappan* phytochemicals should be undertaken to explore their potential cardioprotective effects that could be attributed to xanthine oxidase inhibition, such as the expression of extracellular matrix proteins (collagen and fibronectin), growth factors, and inflammatory and oxidative stress biomarkers.

Numerous studies have demonstrated that *C. sappan* extract and its bioactive compounds can reduce heart transplant and allograft rejection. The extract itself was reported to reduce cell ultrastructural damage and pathological morphology in transplanted hearts by reducing perforin mRNA expression (Zhou et al., 2003), whereas the ethyl acetate extract decreased granzyme B (GrB) mRNA expression, comparable to its positive control, cyclosporine A (Zheng et al., 2008). However, both studies lacked a negative control, a group without any treatment for a comparison. Both perforin and GrB are involved in target cell apoptosis (Voskoboinik et al., 2015), indicating that the compounds exert their beneficial effects by diminishing cell apoptosis. The aqueous extract also suppresses T-lymphocyte activation and increases CD4^+^ CD25^+^ T cells (Yu et al., 2004; Li et al., 2015), contributing to its immunosuppressive effect, hence reducing the rejection rate. The cardioprotective effects could also be attributed to the presence of protosappanin A. At a dose of 25 mg/kg, the compound prolonged heart allograft survival and decreased pathological damage, leading to reduced graft rejection, comparable to cyclosporine A (Wu et al., 2008). The antirejection property of the compound may be due to the diminished inflammation, observed as reduced mRNA expression of NF-κB; suppressed immune response, indicated by decreased interferon-gamma (IFN-γ), interferon-gamma-inducible protein 10 (IP10), and the CD4^+^/CD8^+^ ratio; and lower levels of apoptosis, demonstrated by downregulated GrB and perforin mRNA expression (Wu et al., 2008; Wu et al., 2010). In conclusion, protosappanin A may promote immunosuppression in recipients by targeting the graft’s T cells through inhibition of the NF-κB pathway activation and apoptosis. Further studies should be undertaken to extensively investigate the antirejection effects of protosappanin A, which has the potential to be employed clinically.

## Effects on Vascular Function and Injury

### *Caesalpinia sappan* Extract

Endothelial dysfunction promotes vascular injury by disrupting vascular tone and redox balance, and activating inflammatory responses (Sun et al., 2020). Studies investigating the effects of *C. sappan* extract on vascular function and injury are still lacking, although the extract was demonstrated to protect against brain I/R injury (Wan et al., 2019).

*Caesalpinia sappan* crude extract (10 and 30 μg/ml) had a vasorelaxant effect on precontracted intact rat aortic rings but not denuded rings (Hu et al., 2003). The effects were reduced by N(G)-nitro-l-arginine methyl ester (l-NAME), a nitric oxide synthase (NOS) inhibitor. However, at higher concentrations of the extract (100 μg/ml), the vasorelaxant effect was not affected by denudation or l-NAME treatment (Xie et al., 2000), implying that the effects are both dependent on and independent of the functional endothelium. It is possible that at higher concentrations, the extract may exert its effects by directly stimulating smooth muscle cells rather than by activating the release of nitric oxide, which is primarily synthesized in the endothelium (Cyr et al., 2020). A drawback of the studies (Xie et al., 2000; Hu et al., 2003) was no validated identification of the dried heartwood sappanwood which was obtained from a local store. Therefore, reproducibility of the findings could be questioned.

### Brazilin

**FIGURE 3:**
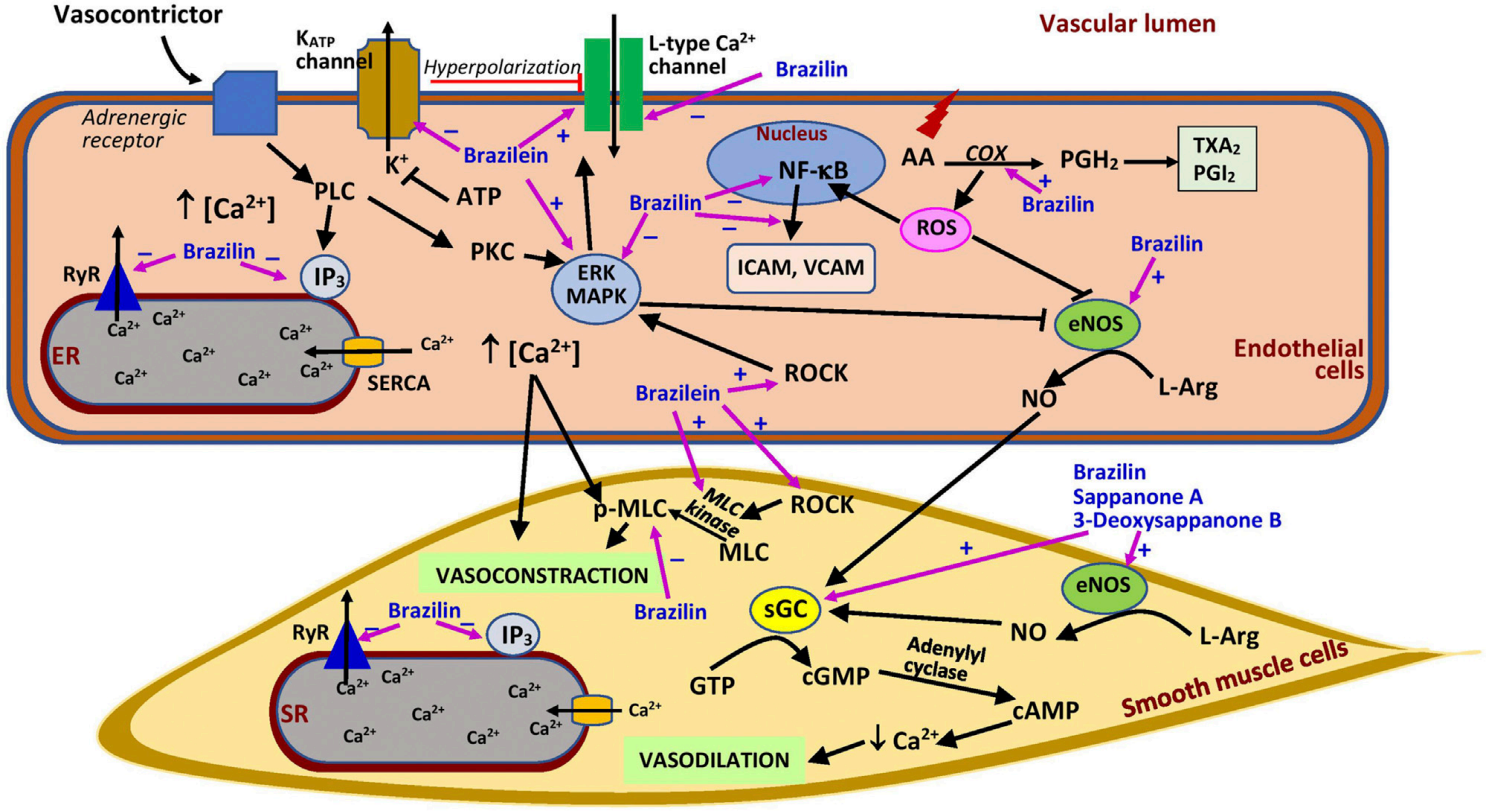
Possible sites of action of *Caesalpinia sappan* bioactive compounds in vascular.AA, arachidonic acid; ATP, adenosine triphosphate; cAMP, cyclic adenosine monophosphate; COX, cyclooxygenase; eNOS, endothelial nitric oxide synthase; ER, endoplasmic reticulum; ERK, extracellular signal-regulated kinase; GTP, guanosine-5′-triphosphate; ICAM, intercellular adhesion molecule; IP3, inositol trisphosphate; K_ATP_ channel, ATP-sensitive potassium channel; L-Arg, l-arginine; MAPK, mitogen activated protein kinase; MLC, myosin light chain kinase; pMLC, phosphorylated myosin light chain; NF-κB, nuclear factor-κB; NO, nitric oxide; PGH_2_, prostaglandin H_2_; PGI_2_, prostacyclin; PKC, protein kinase C; PLC, phospholipase C; ROCK, Rho kinase; ROS, reactive oxygen species; RyR, ryanodine receptor; SERCA, sarcoplasmic/endoplasmic reticulum Ca^2+^ ATPase; sGC, soluble guanylate cyclase; SR, sarcoplasmic reticulum; TXA_2_, thromboxane A_2_; VCAM, vascular cell adhesion molecule; ⊥, suppression; +, stimulates; −, inhibits.

Similar to the crude extract, brazilin exhibited vasorelaxant properties that were dependent on the endothelium at lower concentrations (30 µM) (Hu et al., 2003) but independent of the endothelium at higher concentrations (100 µM) in precontracted intact rat aortic rings (Xie et al., 2000; Yan et al., 2015). However, Hu et al. (2003) demonstrated that the effect of brazilin was only endothelium-dependent and suggested that the relaxing property of the extract at higher concentrations was possibly attributable to the presence of brazilin, which was similar to histamine (50 μM). In the endothelium-dependent vasorelaxation mechanism, brazilin may induce relaxation by decreasing the influx of Ca^2+^ through L-type calcium channels and its release from the sarcoplasmic reticulum via the compound’s effects on RyR2 and inositol trisphosphate (IP_3_) receptors, thus enhancing SERCA activity (Yan et al., 2015), but unfortunately these effects could not be compared with a positive control which was not included in the study (Figure 3). The vasodilating effect of the compound is notably deterred by l-NAME and hemoglobin (a nitric oxide scavenger), indicating that its effect involves the presence of nitric oxide (Hu et al., 2003; Yan et al., 2015). This is further confirmed by its positive effects on nitric oxide synthesis via activation of endothelial NOS (eNOS) (Hu et al., 2003). Following this activation, cyclic guanosine monophosphate (cGMP) is generated from guanosine-5′-triphosphate (GTP) by the action of soluble guanylate cyclase (sGC) in vascular smooth muscle. Brazilin is reported to augment the accumulation of sGC in the rat aorta, and pretreatment with methylene blue, an sGC inhibitor, markedly diminishes the vasodilating effect of brazilin, suggesting a crucial role of sGC in brazilin’s mechanisms of action (Hu et al., 2003; Yan et al., 2015). The increase in cGMP level would enhance the level of cAMP, causing a decline in Ca^2+^ level and finally subsiding the constraction of the blood vessels.

The vasorelaxant effect of brazilin may also involve prostaglandin synthesis, as suggested by its reduced effect with indomethacin, a cyclooxygenase inhibitor, as well as its inhibition of extracellular signal-regulated kinase (ERK) and myosin light chain (MLC) activation (Yan et al., 2015). Cyclooxygenase is an enzyme that synthesizes prostaglandin from arachidonic acid. Prostaglandin itself is a strong vasodilator, which is also converted into products such as thromboxane, a vasoconstrictor, and prostacyclin, a vasodilator (Rouzer and Marnett, 2020). Therefore, brazilin may augment the production of prostaglandin which then promotes vasodilation. By contrast, ERK has an inhibitory effect on eNOS, impairing vasodilation, and MLC causes vasocontraction (Figure 3). Thus, the activation of ERK and MLC plays a crucial role in vascular smooth muscle cell contractile function (Roberts, 2012; Liu and Khalil, 2018). However, it was postulated that the brazilin vasodilatory effect is not mediated by Ca^2+^-dependent K^+^ channel activation (Yan et al., 2015), which is responsible for blood vessel dilation (Hasan and Jaggar, 2018). All these results suggest that brazilin has the potential to act as an agent that promotes vasorelaxation and as a calcium antagonist. However, further studies should explore this potential in depth, especially through *in vivo* investigations in hypertensive animals to ascertain its effects on blood pressure. Future studies should also focus on the ability of brazilin to block calcium channels, and well-designed studies are necessary to compare the activity of brazilin with that of existing calcium channel blockers. Through further research, brazilin has the potential to be developed as a blood pressure-lowering agent.

Brazilin pretreatment (at 50 and 100 μM) also ameliorates vascular inflammation and oxidative stress induced by high glucose in human umbilical vein endothelial cells (HUVEC). High glucose enhances the formation of cell adhesion molecules, i.e., intercellular (ICAM) and vascular adhesion molecules (VCAM) in the cells, which are reduced by brazilin (Jayakumar et al., 2014) (Figure 3). High glucose promotes NF-κB activation, which mediates the inflammatory response by upregulating cell surface expression of adhesion molecules (Morigi et al., 1998). The beneficial effects of the compound are believed to be mediated by its ability to inhibit the activation of ERK, NF-κB, and eNOS triggered by exposure to high glucose (Jayakumar et al., 2014). These findings suggest that brazilin may be useful in the management of diabetes involving microangiopathy. *C. sappan* crude extract has been used to control diabetes in folk medicines (Mekala and Radha, 2015). However, more in-depth research should be carried out for conclusive evidence. Brazilin was also reported to be protective against renal I/R injury by suppressing the NF-κB signaling pathway (Jia et al., 2016).

Platelets are a blood component involved in blood coagulation in response to bleeding due to vascular injury. Brazilin exerted antiplatelet activity in mouse platelets *in vitro* (Ji et al., 2019), whereas Chang et al. (2013) reported brazilin’s ability to stimulate platelet aggregation in human platelets *in vitro*. The discrepancy in these findings could be due to the difference in the platelet source species. Further study is needed to clarify this issue. Chang et al. (2013) demonstrated that brazilin potentiated collagen-induced platelet aggregation *in vitro* at lower concentrations (1–10 μM), whereas at higher concentrations (20–50 μM), it had a direct effect on platelet aggregation. This property associates with its traditional use in treating bleeding gums and uterine bleeding (Mekala and Radha, 2015). The addition of yohimbine (an adrenoceptor antagonist) and a thrombin protease-activated receptor antagonist had no significant effect on brazilin’s properties. However, the addition of caffeic acid phenethyl ester (a collagen receptor antagonist) reduced platelet aggregation. These observations suggest that the platelet-activating property of brazilin does not involve adrenoceptor or protease-activated receptor stimulation; instead, it may be due to the direct activation of collagen receptors. By contrast, it was suggested that brazilin exerts its antiplatelet activity by stimulating protease-activated receptor 4 (Ji et al., 2019). Brazilin is a promising candidate for further research on its potential as a collagen receptor agonist. This property is beneficial in the clinical management of vascular injury that involves blood coagulation.

Other favorable properties of brazilin are the prevention of vascular smooth muscle cell migration and proliferation which was demonstrated in an *in vitro* study (Guo et al., 2013). These vascular events can enhance vascular diseases, such as restenosis and atherosclerosis (Louis and Zahradka, 2010). Brazilin (at 3, 10, and 30 μM) inhibited platelet-derived growth factor (PDGF)-induced vascular smooth muscle cell proliferation by inducing G_0_/G_1_ cell cycle arrest without affecting cell viability. It also downregulated G_0_/G_1_ phase regulatory proteins, namely cyclin E and cyclin-dependent kinase 2 (CDK2), and upregulated p27, but it had no effect on cyclin D. Cyclins D and E, and CDK2 are positive regulators for the G_0_/G_1_ transition, whereas p27 is a negative regulator (Guo et al., 2013). Inhibition of cell migration by brazilin is associated with reduced cellular expression of adhesion molecules (ICAM-1 and VCAM-1) and matrix metalloproteinase-9 (MMP-9). MMPs are mediators of the progression of vascular lesions (Suh et al., 2006). The inhibitory effects of brazilin on both cell proliferation and migration are believed to be linked to the suppression of PDGF receptor β activation, thereby preventing the signaling cascade and leading to the inhibition of ERK1/2, Src, and Akt activation (Guo et al., 2013). ERK1/2 activation promotes cell growth and migration, events that are important in the commencement and development of vascular lesions (Suh et al., 2006), whereas the activation of Src kinases and PI3K/Akt are associated with various cellular events, such as cellular differentiation, proliferation, and cytoskeletal reorganization (Sayeski and Ali, 2003; Zhang et al., 2020). Despite its good findings, a shortcoming of the study (Guo et al., 2013) is an absence of a positive control group. Taken together, brazilin has the potential to prevent atherosclerosis and restenosis, and these effects warrant further *in vivo* studies.

### Brazilein

Different from brazilin, its oxidized form, brazilein (at 100 μM), promotes vasocontraction in rat thoracic aortic rings, comparable to caffeine (20 mM) but lesser than phenylephrine (10 μM). The effect is not endothelial-dependent and does not involve the stimulation of α- and β-adrenoceptors, muscarinic receptors, or angiotensin II type 1 receptors (Shen et al., 2008). However, its effects are significantly weakened by the addition of nimodipine and diltiazem (L-type Ca^2+^ channel blockers) and pinacidil (a potassium channel opener), indicating that the vasocontraction effect of brazilein is dependent on Ca^2+^ influx (Shen et al., 2008). Extracellular Ca^2+^ entry is required for the contraction of vascular smooth muscle cells. Pinacidil increased potassium efflux, leading to hyperpolarization, which then reduced Ca^2+^ influx (Tinker et al., 2018) (Figure 3). The reduction in Ca^2+^ entry diminishes the effects of brazilein. The effect of brazilein on blood vessels was not associated with the activation of protein kinase C (PKC) or IP_3_ receptor, demonstrated by the lack of effect observed with the addition of their respective inhibitors (Shen et al., 2008). However, the co-administration of inhibitors of MLC kinase (MLCK), Rho-kinase (ROCK), and ERK decreased the vasocontraction property of the compound. Collectively, these findings suggest that brazilein most likely exerts vasocontraction via the activation of the L-type calcium channel and the involvement of ROCK, MLCK, and ERK. More studies, especially *in vivo* studies, are needed to confirm the effects seen in the *in vitro* setting.

### Other Compounds

Few studies to date have investigated other bioactive compounds isolated from *C. sappan* for their potential role in vascular injury. Hematoxylin (at 10, 30, and 100 µM) from the heartwood also demonstrated vasorelaxant activity in precontracted intact rat aortic rings, but the effects were reduced in denuded rings and these observations were not compared with a positive control (Xie et al., 2000). The effects were also diminished by l-NAME, suggesting the involvement of nitric oxide in its effect. He et al. (2009) reported vasorelaxant effects of (E)-3-(3,4-dihydroxybenzylidene)-7-hydroxychroman-4-one, sappanone B, and 3-deoxysappanone B in endothelium-intact and endothelium-denuded aortic rings, suggesting an independent effect of the presence of endothelium (Figure 3). However, the vasorelaxant activity of the compounds was diminished in the presence of an eNOS inhibitor and an sGC inhibitor, indicating that the compounds exerted their effects via the nitric oxide-cGMP pathway (He et al., 2009). Nonetheless, it is not yet confirmed whether these compounds elicit the effect by acting directly or indirectly on the vascular muscarinic receptors. Further studies should be conducted to clarify this. On the other hand, 1-hydroxy-7-methylxanthone, 1,7-dihydroxyxanthone, butein, and sappanone A were demonstrated to significantly inhibit lipopolysaccharide-induced nitric oxide production *in vitro* (Zhao et al., 2014). A high level of nitric oxide in blood vessels is beneficial because of its vasodilating effect (Kamisah et al., 2017), but its synthesis is also enhanced under oxidative stress, which could be harmful because of its ability to form peroxynitrite radicals (Kamisah et al., 2016). Therefore, the ability of these *C. sappan* phytochemicals to suppress nitric oxide production following exposure to stress is advantageous.

Sappanchalcone also possesses antiplatelet activity. Other compounds, such as 3-deoxysappanone B, caesalpin J. epicaesalpin J, episyringaresinol, methylesappanol, and protosappanin A isolated from the plant were also screened and found to have negligible effects (Ji et al., 2019). However, the possible mechanism of action of the sappanchalcone was not elucidated in the study. The compound may manifest its property via the same mechanism as brazilin, but this postulation has yet to be confirmed. Its effects on other clotting factors, such as prothrombin, activated factor IX, and plasminogen activator, should also be studied.

## Conclusion and Directions for Future Study

This review shows that *C. sappan* Linn. contains many bioactive compounds, such as brazilin, brazilein, and sappanone A, that should be further studied and developed as potential candidates for treating cardiovascular problems, particularly myocardial infarction, cardiac remodeling, and hypertension. However, well-designed studies with appropriate controls (negative and positive) and a good range of doses to date are still at a preliminary stage. *In vivo* studies to confirm the activities seen in *ex vivo* and *in vitro* studies are still lacking. The effects of *C. sappan* bioactive compounds on myocardial calcium handling proteins, mitochondrial function, PI3K/Akt/mammalian target of rapamycin (mTOR), and cellular mechanotransduction, as well as the renin–angiotensin–aldosterone system, should be explored to better understand their mechanistic pathways. Certain genes involved in the pathogenesis of hypertension—Alb, Chrm2, Xirp1, Kcnq1, Slc5a7, Kcnh1, Ache, Crlf1 and Galr2— should also be studied. To progress to clinical trials, the safety of the compounds for administration to humans should also be determined.
